# Easing Cash Assistance Rules and Breastfeeding

**DOI:** 10.1001/jamahealthforum.2025.2999

**Published:** 2025-08-08

**Authors:** Emily C. Dore, Daniel F. Collin, David W. Rothwell, Rita Hamad

**Affiliations:** 1 Department of Social and Behavioral Sciences, T. H. Chan School of Public Health, Harvard University, Boston, Massachusetts; 2 Human Development and Family Sciences, College of Health, Oregon State University, Corvallis

## Abstract

**Importance:**

Temporary Assistance for Needy Families (TANF) is a major federal cash assistance program for low-income families, but the program has strict eligibility criteria, meager cash benefits, and cumbersome administrative burdens that limit its effect. However, during the COVID-19 pandemic state TANF programs implemented policy changes to expand program access, including relaxed work requirements and additional income. Few studies have examined the association of these policy changes with health outcomes.

**Objective:**

To analyze the association between TANF policy changes during the COVID-19 pandemic and breastfeeding initiation and duration.

**Design, Setting, and Participants:**

This study used serial cross-sectional national data from the Pregnancy Risk Assessment Monitoring System (2017-2020) and a quasi-experimental difference-in-differences analysis to examine whether TANF policy changes affected breastfeeding behaviors among likely TANF participants compared with likely nonparticipants. Multivariable linear regression analyses were conducted that adjusted for individual and state-level covariates. The study was conducted between November 2024 and May 2025.

**Exposures:**

Gave birth in a state that implemented TANF policy changes (eg, 1-time cash payments, waiving in-person interviews and work requirements, and extending time limits).

**Main Outcomes and Measures:**

Self-reported measures of whether breastfeeding was initiated and breastfeeding duration in weeks.

**Results:**

The sample included 138 700 individuals; the likely TANF participants were less likely to be White (~33%) or married (~37%), were younger (~29% were 20-24 years of age), had lower incomes (~$30 000), and were more likely to have 2 or more previous births (~39%) compared with the likely nonparticipants. The TANF policy changes that expanded eligibility and decreased administrative burdens were associated with increased breastfeeding initiation and duration; eg, waiving sanctions was associated with increased breastfeeding initiation (2.59 percentage points [95% CI, 0.13-5.06 percentage points]) and automatic recertification was associated with increased breastfeeding duration (0.49 weeks [95% CI, 0.04-0.94 weeks]). Providing 1-time cash payments was also associated with increased breastfeeding duration (1 week [95% CI, 0.42-1.58 weeks]). These positive associations were found across all racial and ethnic groups, although some 95% CIs included the null.

**Conclusions and Relevance:**

In this quasi-experimental study, state TANF policies that expanded eligibility, increased cash payments, and decreased administrative burdens were associated with increased breastfeeding initiation and duration. These findings inform active policymaking on antipoverty policies.

## Key Points

**Question:**

Did changes to the Temporary Assistance for Needy Families (TANF) program during the COVID-19 pandemic affect breastfeeding initiation rates and breastfeeding duration?

**Findings:**

In this quasi-experimental study of 138 700 individuals, TANF policy changes that expanded eligibility, increased cash payments, and decreased administrative burdens were associated with increased breastfeeding initiation and duration.

**Meaning:**

Increasing TANF access may facilitate breastfeeding and improve maternal and child health; these findings inform active policymaking on antipoverty policies.

## Teaser

This quasi-experimental study assesses the association between Temporary Assistance for Needy Families policy changes during the COVID-19 pandemic and breastfeeding initiation and duration among likely TANF participants compared with likely nonparticipants.

## Introduction

Poverty is associated with poorer health due to increased stress and less access to health-related resources, including quality housing, healthy foods, and health care.^1^ Poverty rates increase around pregnancy because of higher medical and nutritional needs and more barriers to work; this makes income support especially important during this period.^2^ As a result, many parents rely on the social safety net, including the Temporary Assistance for Needy Families (TANF) program.^3^ Since welfare reform in the mid-1990s, the TANF program has prioritized transitioning participants from welfare to work using work requirements, sanctions for nonadherence with work requirements, time limits on participation length, and other levers to incentivize work. Due to these policy components, the TANF program has a limited reach, providing cash benefits to only about 20% of families experiencing poverty.^4^

Welfare reform also devolved administration of the program (previously known as Aid to Families with Dependent Children) to individual states, leading to variation in programmatic components such as the total cash benefits, income eligibility, and exemptions from work requirements.^4,5^ For example, monthly benefits in 2023 for a 3-person family ranged from $204 in Arkansas to $1243 in New Hampshire.^6^ Evidence is mixed on the health effects of the TANF program, although studies often differ on which components of the TANF program they study, the sample chosen as eligible for the TANF program, and the health outcomes.^7,8,9^ During the COVID-19 pandemic, states implemented TANF policy changes in response to the public health and unemployment crises; the TANF policy changes were intended to expand resources and eligibility while reducing administrative burdens.^10^ The policy changes included 1-time cash payments; waiving work requirements; waiving in-person interviews; waiving or pausing sanctions; extending time limits for the program; and automatically recertifying benefits.^11^

The current study examined the association of these TANF policies with breastfeeding behaviors as one health outcome potentially affected by these shifts. The American Academy of Pediatrics recommends infants be breastfed exclusively until age 6 months.^12^ However, breastfeeding rates in the US are low; about 87% of women reported ever breastfeeding and 48% reported exclusively breastfeeding at 3 months.^13^ In addition, there are socioeconomic and racial disparities in breastfeeding. Individuals with lower education levels and Black individuals are the least likely to breastfeed.^13^

Breastfeeding is associated with better maternal and child health, including lower rates of hypertension and breast cancer for the person breastfeeding^14,15^ and decreased infant mortality.^16^ About 61% of pregnant people intend to breastfeed exclusively, although only 51% report doing so.^17^ Factors such as occupation, education, stress, and income are associated with breastfeeding initiation and duration.^18,19,20^ The need to return to work is a particularly significant barrier in the US because there is no national paid leave policy.^21,22^ Different TANF policies may affect breastfeeding by influencing parents’ flexibility around returning to work after birth (eg, due to work requirements or benefit generosity).

Previous work on the TANF program and breastfeeding is scarce and inconclusive. One study,^23^ which examined state stringency of work requirements after welfare reform, found that breastfeeding rates were 3.1 percentage points lower in states with the most stringent requirements. Another study compared individuals based on whether the parents were exempt from working during their child’s first year of life, and found these exemptions were not associated with breastfeeding.^24^ The 2 studies were published in 2003 and 2011 and examined TANF policies focused only on work requirements.^23,24^

Our study updates this literature by assessing the association between breastfeeding and TANF policies beyond work requirements, and during a more recent period that captures a different political, economic, and social climate. Specifically, we hypothesize that the pandemic-era TANF policy changes that increased eligibility and resources and decreased administrative burdens resulted in increased breastfeeding initiation and duration (eFigure 1 in Supplement 1). Although individual states have mostly reverted back to prepandemic TANF policies, some states have made other recent changes, including increasing cash benefit levels.^6^ The generalizability of our study’s findings is limited due to the context of the COVID-19 pandemic, but this study speaks to the potential effects of these types of policies.

## Methods

The current study examined the association between COVID-19 pandemic–era TANF policy changes and breastfeeding among likely TANF participants (as defined by individuals’ education level, a proxy often used for TANF eligibility) compared with likely TANF nonparticipants.^25,26,27^ This study used deidentified data and was deemed exempt from obtaining patient consent by the institutional review board of Harvard University. The Strengthening the Reporting of Observational Studies in Epidemiology (STROBE) reporting guideline^28^ was used. The study was conducted between November 2024 and May 2025.

### Data

This quasi-experimental study used serial cross-sectional national data from the Pregnancy Risk Assessment Monitoring System (PRAMS) for 2017-2020. The year range captures prepandemic (pre-March 2020) to pandemic-era (March-December 2020) outcomes. PRAMS is a collaboration between state, territorial, and local health departments and the US Centers for Disease Control and Prevention.^29^ Each participating site annually surveys 1000 to 3000 individuals who recently had a live birth and asks about perinatal health and health behaviors, health care use, and early infant development.

We included individuals with singleton births of infants with gestational ages of 22 to 44 weeks. The sample included 138 700 individuals who had nonmissing data for at least 1 breastfeeding outcome and who had complete data for the covariates (eFigure 2 in Supplement 1). The individuals were from 43 states plus Washington, DC. Seven states did not provide data for the study period and were excluded (additional information appears in the eMethods in Supplement 1).

### Variables

#### Exposure

The exposure variables were state TANF policy changes that would plausibly affect breastfeeding. The variables were drawn from a publicly available database that captured information on changes to TANF state programs during the pandemic.^30^ The exposure variables included 1-time cash payments to TANF participants and low-income participants not otherwise eligible for the TANF program, decreased administrative burdens (eg, waiving in-person interviews, waiving or pausing sanctions), and expanded eligibility (eg, not counting participation during the COVID-19 pandemic toward the TANF program time limits) (eTable in Supplement 1).

The states varied in whether and when they implemented the TANF policy changes; most were enacted in March or April 2020 and were in place through December 2020. We considered someone exposed to the TANF policy changes if the birth date fell after the policy change was implemented and before it ended, if applicable. The study period was limited to 2017-2020 because the TANF policy database captured exposure data most reliably through December 2020.

#### Outcomes

We examined 2 self-reported outcomes. The first outcome was whether the individual ever breastfed (ie, breastfeeding initiation). The second outcome was the number of weeks the individual breastfed (ie, breastfeeding duration, which was coded as 0 for those who never breastfed). Additional information appears in the eMethods in Supplement 1.

#### Covariates

The covariates included maternal age, race and ethnicity, marital status, parity, and family income during the year before birth. Race and ethnicity categories were self-reported and included Hispanic and non-Hispanic Asian/Pacific Islander, Black, White, or other race. The non-Hispanic other race group consisted of individuals who identified as American Indian or Alaska Native, multiracial, or other. The latter was heterogeneous but small sample sizes precluded more granular analyses. The covariates also included the following time-varying state-level characteristics based on birth date and state of residence: dichotomous variables representing the presence of a state paid family leave or child tax credit policy and continuous variables for the earned income tax credit rate, poverty rate, and unemployment rate.^31,32,33,34^

### Primary Analysis

We calculated sample descriptive statistics before vs during the pandemic (ie, before March 2020 vs March-December 2020) and based on likelihood of TANF participation. Because PRAMS does not ask about TANF participation, we used maternal education as a proxy for eligibility, which is similar to other studies of the TANF program.^25,26,27^ Specifically, we considered individuals with a high school education or less to be likely TANF participants and individuals with greater than a high school education to be likely nonparticipants. Notably, 90% of TANF participants have this level of education (≤high school) compared with 28% of the general population.^35,36^

We estimated the association of TANF policy changes with breastfeeding using a quasi-experimental triple-difference design.^37^ A traditional difference-in-differences analysis compares outcomes before and after an intervention among individuals residing in states that implemented the policy (“treated”) vs individuals residing in states that did not implement the policy (“control”). A triple-difference analysis further compares individuals likely to be TANF participants vs those likely to be nonparticipants. The coefficient of interest in this analysis involved a triple-interaction term combining 3 indicator variables. The first indicator variable was whether the state implemented the TANF policy change; the second was whether the birth occurred before or after the policy change was implemented; and the third was whether the individual was likely to be a TANF participant.

Models were used to test for the association of 1 policy change at a time using multivariable regression models (adjusted for the covariates listed above, other TANF policy changes of interest in that state and month, and state and month fixed effects). For binary outcomes, these models represent linear probability models, and the coefficients are interpreted as a percentage point change. The SEs were clustered at the state level. We did not apply survey weights to the main analyses because our goal was to estimate causal effects.^38,39^ However, a sensitivity analysis was performed that applied survey weights for comparison (additional information appears in the eMethods in Supplement 1).

### Secondary Analyses

We created a composite variable representing the number of TANF policy changes implemented in each state to capture an overall policy context,^40^ and examined the association of this composite variable with breastfeeding comparing individuals likely to be TANF participants vs those likely to be nonparticipants. Next we estimated the association between individual policy changes and the composite policy variable with breastfeeding stratified by race and ethnicity. Studies have found racial discrimination in the welfare system (eg, Black and Hispanic TANF participants were sanctioned more frequently than White participants^41^), suggesting the association of the policy changes with breastfeeding may vary by race and ethnicity.

We further conducted sensitivity analyses to test the robustness of the identification strategy by using a different proxy for TANF participation. Instead of using education level, the treated group was defined as having a family income of $25 000 or less and the control group was defined as having a family income greater than $25 000. Second, the sample was limited to individuals with a family income of $75 000 or less, comparing the original education groups (≤high school vs >high school) and income groups (≤$25 000 vs >$25 000).

### Model Assumptions

One assumption of difference-in-differences designs is that pre-post trends among treated and control groups would be similar (parallel) if the intervention had not occurred, which is known as the parallel trends assumption. Because this counterfactual cannot be empirically tested, we assessed trends among the treated and control groups before the TANF policy change by conducting event-study analyses comparing individuals in states without a policy change vs individuals in states with a policy change.

## Results

### Sample Characteristics

The sample included 138 700 individuals. The likely TANF participants were less likely to breastfeed (~80%) and had a shorter breastfeeding duration (~9 weeks) compared with the likely nonparticipants. The likely TANF participants were also less likely to be White (~33%) or married (~37%), were younger (~29% were 20-24 years of age), had lower incomes (~$30 000), and were more likely to have 2 or more previous births (~39%) and Medicaid coverage (~71%) compared with the likely nonparticipants (Table).

**Table.** Outcomes and Characteristics From the Pregnancy Risk Assessment Monitoring System From January 2017 to December 2020 (N = 138 700)

Outcomes and characteristics	January 2017-February 2020^a^		March 2020-December 2020^a^^,^^b^	
	Likely nonparticipants^c^	Likely TANF participants^d^	Likely nonparticipants^c^	Likely TANF participants^d^
**Outcomes**				
No. of observations	72 573	37 687	18 890	9550
Ever breastfed	92.7	79.4	93.2	80.8
Breastfeeding length, mean (SD), wk	12.5 (7.3)	9.0 (8.3)	12.9 (7.3)	9.4 (8.4)
**Maternal characteristics**				
Education level				
Less than high school	0	31.2	0	28.7
High school	0	68.8	0	71.3
Some college	44.8	0	42.8	0
College	55.2	0	57.2	0
Family income, mean (SD), $^e^	65 577 (32 845)	29 215 (20 166)	68 895 (32 223)	31 836 (22 183)
Race and ethnicity				
Hispanic	11.0	26.4	12.1	25.4
Non-Hispanic				
Asian/Pacific Islander	8.4	3.7	10.1	4.5
Black	15.3	23.8	14.5	22.8
White	57.0	33.9	53.5	32.7
Other race^f^	8.2	12.3	9.7	14.7
Married	73.7	37.6	73.4	36.9
Age group, y				
≤17	0	2.7	0	2.2
18-19	0.7	7.3	0.6	6.7
20-24	11.5	29.4	10.5	29.2
25-29	29.5	29.0	28.3	28.4
30-34	35.7	19.6	36.9	20.5
35-39	18.6	9.5	19.5	10.0
≥40	4.0	2.5	4.2	2.9
Parity				
No previous births	42.1	32.9	43.4	33.8
1 Previous birth	33.8	28.1	33.0	27.3
≥2 Previous births	24.1	38.9	23.7	38.9
Medicaid	29.3	71.5	28.7	71.0
**State characteristics**				
Paid family leave	9.1	7.3	9.9	8.1
Child tax credit	8.1	8.0	7.4	5.9
Earned income tax credit rate, mean (SD)	0.1 (0.1)	0.1 (0.1)	0.1 (0.1)	0.1 (0.1)
Poverty rate, mean (SD)	10.7 (3.1)	11.3 (3.3)	10.6 (2.7)	10.9 (2.8)
Unemployment rate, mean (SD)	4.0 (1.2)	4.1 (1.2)	7.4 (1.8)	7.2 (1.7)
No. of TANF policy changes, median (IQR)	NA	NA	3 (1-3)	2 (1-3)

Abbreviations: NA, not applicable because changes were implemented during COVID-19 pandemic; TANF, Temporary Assistance for Needy Families.

^a^ Data are expressed as percentages unless otherwise indicated.

^b^ Dates reflect COVID-19 pandemic period.

^c^ This category reflects unlikely participation (education level >high school).

^d^ This category reflects likely participation (education level ≤high school).

^e^ For the year before birth.

^f^ American Indian or Alaska Native, multiracial, or other.

### Model Assumptions

The event-study results showed no difference in breastfeeding outcomes comparing individuals in states with TANF policy changes vs individuals in the states without policy changes before the COVID-19 pandemic (eFigures 3-4 in Supplement 1). These results provided reassurance of the validity of the parallel trends assumption.

### Association Between the TANF Policy Changes and Breastfeeding

**Figure 1.**
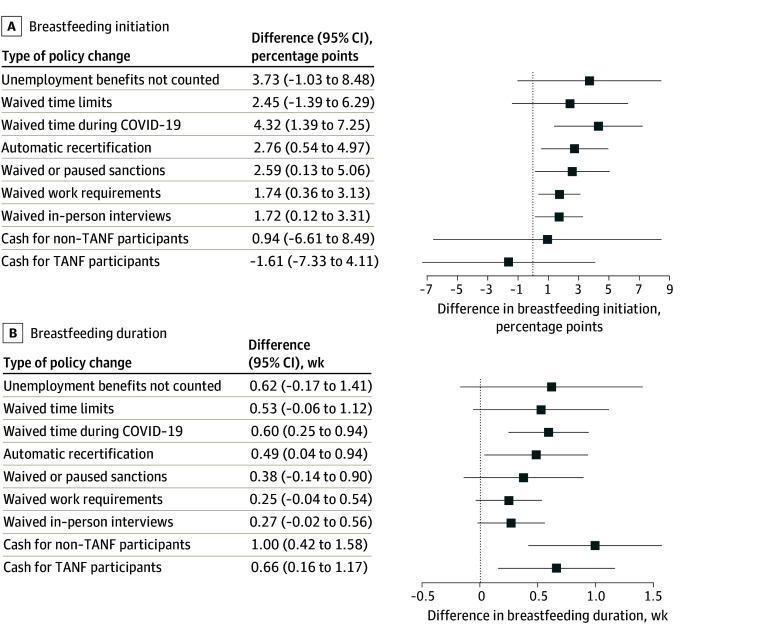
Overall Associations Between Policy Changes for the Temporary Assistance for Needy Families (TANF) Program During the COVID-19 Pandemic and the Rates of Breastfeeding From January 2017 to December 2020 In A, the sample size was 138 435. In B, the sample size was 136 006. The results were derived from a triple-differences approach that measured the difference in outcomes between likely TANF participants (individuals with an education level ≤high school) vs likely nonparticipants (individuals with an education level >high school) in states that had the TANF policy change during the COVID-19 pandemic vs states that did not have the policy change. Each model adjusted for the other 8 policy changes, maternal and state-level covariates, fixed effects for state and month, and clustered SEs at the state level. The whiskers indicate 95% CIs.

In the main triple-difference analysis, many policy changes were associated with increased breastfeeding initiation and duration (Figure 1).

The following TANF policy changes were associated with increased breastfeeding initiation: waiving time limits during the COVID-19 pandemic (4.32 percentage points [95% CI, 1.39-7.25 percentage points]), automatic recertification (2.76 percentage points [95% CI, 0.54-4.97 percentage points]), waiving sanctions (2.59 percentage points [95% CI, 0.13-5.06 percentage points]), waiving work requirements (1.74 percentage points [95% CI, 0.36-3.13 percentage points]), and waiving in-person interviews (1.72 percentage points [95% CI, 0.12-3.31 percentage points]).

The following TANF policy changes were associated with increased breastfeeding duration: waiving time limits during the COVID-19 pandemic (0.60 weeks [95% CI, 0.25-0.94 weeks]); automatic recertification (0.49 weeks [95% CI, 0.04-0.94 weeks]); and providing 1-time cash payments to low-income individuals not previously enrolled in TANF (1 week [95% CI, 0.42-1.58 weeks]) and to TANF participants (0.66 weeks [95% CI, 0.16-1.17 weeks]).

### Secondary Analyses

**Figure 2.**
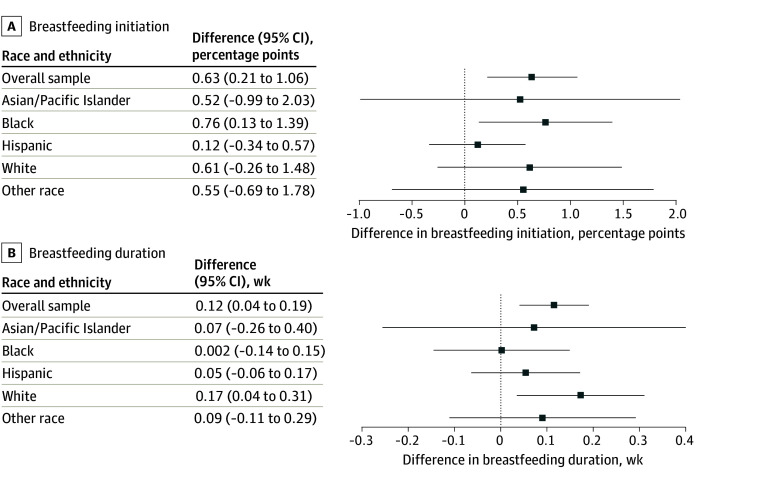
Associations Between the Number of Policy Changes for the Temporary Assistance for Needy Families (TANF) Program During the COVID-19 Pandemic and the Rates of Breastfeeding Overall and Stratified by Race and Ethnicity The results were derived from a multivariable regression that measured the difference in outcomes between likely TANF participants (education level of ≤high school) and likely nonparticipants (education level >high school) based on an index of TANF policy changes during the COVID-19 pandemic as the exposure. Each model adjusted for maternal and state-level covariates, fixed effects for state and month, and clustered SEs at the state level. The whiskers indicate 95% CIs. In A, the overall sample size was 138 435 and the sample size was 9830 for Asian/Pacific Islander race; 24 955 for Black race; 22 600 for Hispanic ethnicity; 67 273 for White race; and 13 777 for other race. In B, the overall sample size was 136 006 and the sample size was 9673 for Asian/Pacific Islander race; 24 461 for Black race; 22 276 for Hispanic ethnicity; 66 086 for White race; and 13 510 for other race. The other race group consisted of individuals who identified as American Indian or Alaska Native, multiracial, or other.

In states where more of the relevant TANF policy changes were passed, breastfeeding initiation increased by 0.63 percentage points (95% CI, 0.21-1.06 percentage points) and breastfeeding duration increased by 0.12 weeks (95% CI, 0.04-0.19 weeks) (Figure 2). In the results stratified by race and ethnicity, breastfeeding initiation increased by 0.76 percentage points (95% CI, 0.13-1.39 percentage points) among Black individuals and breastfeeding duration increased by 0.17 weeks (95% CI, 0.04-0.31 weeks) among White individuals. There were no associations for any of the other racial and ethnic groups, although the coefficients were also positive.

More TANF policy changes, including waiving time limits, were associated with increased breastfeeding initiation among Black individuals and individuals of other race (eFigure 5 in Supplement 1). More TANF policy changes were associated with increased breastfeeding duration for Hispanic individuals, White individuals, and individuals of other race, including waiving in-person interviews for White individuals and temporary cash payments for Hispanic individuals and individuals of other race (eFigure 6 in Supplement 1). There were fewer associations among Asian/Pacific Islander and Hispanic individuals.

**Figure 3.**
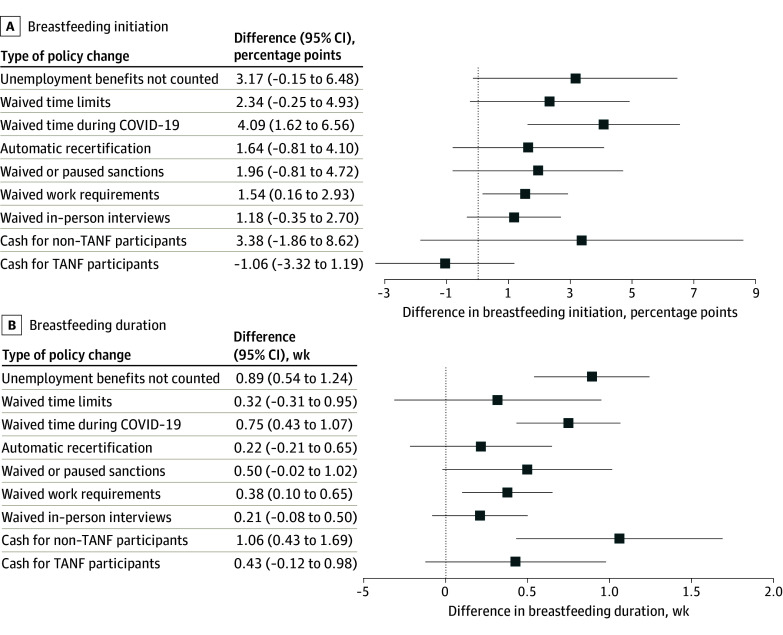
Association Between the Amount of Policy Changes for the Temporary Assistance for Needy Families (TANF) Program During the COVID-19 Pandemic and the Rates of Breastfeeding Using an Alternate Income Identification Strategy to Identify Likely TANF Participants In A, the overall sample size was 138 435. In B, the overall sample size was 136 006. The results were derived from a triple-differences approach that measured the difference in outcomes between likely TANF participants (individuals with a yearly income of ≤$25 000) and likely nonparticipants (individuals with a yearly income >$25 000) in states that had TANF policy changes during the COVID-19 pandemic vs states that did not have policy changes. Each model adjusted for 8 other TANF policy changes, maternal and state-level covariates, fixed effects for state and month, and clustered SEs at the state level. The whiskers indicate 95% CIs.

Although slightly attenuated, the findings were similar to the main results when using different proxies to define likely TANF participation (Figure 3 and eFigures 7-8 in Supplement 1) and when the analyses included weights (eFigure 9 in Supplement 1).

## Discussion

Although changes to the TANF program are infrequent, the policy changes implemented during the COVID-19 pandemic provided an opportunity to assess the association between TANF policy changes and breastfeeding outcomes. This study found that increasing access to cash benefits while decreasing administrative burdens was associated with increased breastfeeding initiation and duration for individuals with a high school education or less, who are more likely to be TANF participants. The findings were robust to sensitivity analyses.

The results from the current study align with a study^23^ that found more restrictive work requirements decreased breastfeeding initiation after welfare reform. However, the results from the current study conflict with another study^24^ that found no association between TANF work exemptions and breastfeeding; however, that study examined a different welfare policy (work requirement exemptions based on the ages of the TANF participants’ children) and identified the treated group as single mothers (not limited by education level), which may explain the different findings. Improving the ability to link health data with TANF administrative data would potentially decrease discrepancies across studies using different identification strategies and better allow researchers to identify the health effects of US safety net policies on actual program participants.^42^

The current study results align with other studies^21,43,44,45^ that found US policies (such as state paid leave) that provide resources while not requiring work after birth can increase breastfeeding rates. The current study results are similar to those found in evaluations of paid leave policies in other countries^46,47^ that have similar percentages of women in the labor force but have more weeks of paid leave and longer average breastfeeding durations. Breastfeeding duration also increased dramatically during pandemic-era shelter-in-place policies,^43^ suggesting that providing opportunities for breastfeeding may facilitate desired breastfeeding behaviors. Similarly, policies that provide material or health-related resources have also been found to increase breastfeeding, including the earned income tax credit^48^ and the Affordable Care Act.^49^

We found differential associations between policy changes and breastfeeding across racial and ethnic groups. Participation in the TANF program increased at the beginning of the COVID-19 pandemic and was at least partially due to the TANF policy changes examined in this study.^50^ However, preliminary work^51^ found racial and ethnic inequalities in TANF participation after March 2020. Asian, Hispanic, Native Hawaiian/Pacific Islander, and White individuals had increased participation at the onset of the COVID-19 pandemic, but Black individuals did not have increased participation.^51^ The differences in the TANF participation rates may at least partially explain the association between the policy changes and breastfeeding among these groups. However, there were also positive associations between TANF policy changes and the initiation of breastfeeding for Black individuals. These findings suggest decreasing administrative burdens likely improves access to the TANF program and associated health outcomes for Black participants, who are in a racial group that has faced discrimination and racialized burdens in the welfare system.^41,52^ On the other hand, the null associations across some groups may reflect the smaller sample sizes resulting from stratified analyses (coefficients were almost all positive), but the 95% CIs included the null. Future studies should replicate this analysis in larger datasets.

Alternatively, null findings among Hispanic and Asian/Pacific Islander individuals may be because of immigration-related barriers to TANF participation. Welfare reform in the 1990s restricted immigrant access to the TANF program by requiring individuals to have lived in the US for at least 5 years,^53,54^ resulting in decreased immigrant participation.^55^ In addition, in times of increased anti-immigrant sentiment (such as during the COVID-19 pandemic), immigrants may be even less likely to access the safety net because of deportation fears or other adverse consequences.^56^

For those who can access the TANF program, many do so immediately after their first birth.^57,58^ However, because TANF policies vary across states, the difficulty of meeting eligibility requirements depends on location. For example, some states waive parental work requirements based on the child’s age, but as of 2022, 6 states had no such exceptions,^59^ potentially leading to more low-income parents working right after birth.^60^ Although some jobs offer flexibility to facilitate breastfeeding, supportive workspaces are less available to low-income and Black and Hispanic workers.^61,62^

There have been other recent changes to the TANF program. For example, many states have increased cash benefit amounts, whereas other states have repealed their family cap policies (which disallow an increase in benefits if an individual has another child while participating in the TANF program).^63^ Until recently, the TANF benefit levels in many states had not increased since welfare reform in the 1990s and they have not kept pace with inflation.^64^ Most of the recent increases represent less than a 10% increase from the previous year. However, Kentucky doubled their monthly benefit amount from $262 in 2022 to $524 in 2023 for a single-parent family of 3.^6^ Our results show that even a 1-time extra cash benefit disbursement can increase breastfeeding duration. Future studies should examine whether more recent cash increases have affected TANF participants’ health.

At present, most states have reversed the TANF policy changes, but some states are implementing other policy changes and the health effects from these changes should be evaluated. With increasing responsibility for shaping policies, and in the absence of major federal investments,^65^ states are likely to play a considerable role in shaping maternal and child health.

### Limitations

This study has limitations. First, as in most national surveys, PRAMS does not ask about participation in the TANF program, so we used education level (≤high school vs >high school) as a proxy, resulting in misclassification of some individuals. However, this technique is commonly used in TANF policy evaluations and in studies of other US safety net policies, given the virtual impossibility of linkages with administrative data on program participation.^42^

Second, another challenge was possible confounding by co-occurring policies.^66,67,68^ We adjusted for a number of time-varying policies (eg, paid leave) as well as state and month fixed effects to account for time-invariant state factors and secular trends; nevertheless, this is a limitation of all difference-in-differences analyses.

Third, we were limited in studying some policy changes when they were implemented by only a few states and when states lacked PRAMS data. Nonetheless, we examined 9 different TANF policy changes.

## Conclusions

In this quasi-experimental study, state TANF policies that expanded eligibility, increased cash payments, and decreased administrative burdens were associated with increased breastfeeding initiation and duration. These findings inform active policymaking on antipoverty policies.

## Notes

Supplement 1.
eMethods
**eFigure 1.** Conceptual diagram linking TANF policy changes to breastfeeding outcomes **eFigure 2.** Sample flowchart, Pregnancy Risk Assessment Monitoring System **eFigure 3.** Event study plots for each TANF policy change and breastfeeding initiation **eFigure 4.** Event study plots for each TANF policy change and breastfeeding duration **eFigure 5.** Association between TANF policy changes and breastfeeding initiation by race and ethnicity **eFigure 6.** Association between TANF policy changes and breastfeeding duration by race and ethnicity **eFigure 7.** Association between TANF policy changes and breastfeeding, alternate identification strategy, restricting sample to income level of $75,000 or less and comparing by education level **eFigure 8.** Association between TANF policy changes and breastfeeding, alternate identification strategy, restricting sample to income level of $75,000 or less and comparing by income level **eFigure 9.** Association between TANF policy changes and breastfeeding with survey weights **eTable.** Details on policy changes for the Temporary Assistance for Needy Families program during the COVID-19 pandemic
eReferences

Supplement 2.
Data sharing statement
