# Supplementary material for: Easing Cash Assistance Rules and Breastfeeding
Source: JAMA Health Forum. 2025 Aug 8;6(8):e252999. doi: 10.1001/jamahealthforum.2025.2999 (PMC12334963; doi:10.1001/jamahealthforum.2025.2999)
Supplement: Supplement 1. — eMethods eFigure 1. Conceptual diagram linking TANF policy changes to breastfeeding outcomes eFigure 2. Sample flowchart, Pregnancy Risk Assessment Monitoring System eFigure 3. Event study plots for each TANF policy change and breastfeeding initiation eFigure 4. Event study plots for each TANF policy change and breastfeeding duration eFigure 5. Association between TANF policy changes and breastfeeding initiation by race and ethnicity eFigure 6. Association between TANF policy changes and breastfeeding duration by race and ethnicity eFigure 7. Association between TANF policy changes and breastfeeding, alternate identification strategy, restricting sample to income level of $75,000 or less and comparing by education level eFigure 8. Association between TANF policy changes and breastfeeding, alternate identification strategy, restricting sample to income level of $75,000 or less and comparing by income level eFigure 9. Association between TANF policy changes and breastfeeding with survey weights eTable. Details on policy changes for the Temporary Assistance for Needy Families program during the COVID-19 pandemic eReferences [file jamahealthforum-e252999-s001.pdf]

Supplemental Online Content

Dore EC, Collin DF, Rothwell DW, Hamad R. Easing cash assistance rules and breastfeeding. *JAMA Health Forum*. Published online August 8, 2025. doi:10.1001/jamahealthforum.2025.2999

eMethods

eFigure 1. Conceptual diagram linking TANF policy changes to breastfeeding outcomes

eFigure 2. Sample flowchart, Pregnancy Risk Assessment Monitoring System

eFigure 3. Event study plots for each TANF policy change and breastfeeding initiation

eFigure 4. Event study plots for each TANF policy change and breastfeeding duration

eFigure 5. Association between TANF policy changes and breastfeeding initiation by race and ethnicity

eFigure 6. Association between TANF policy changes and breastfeeding duration by race and ethnicity

eFigure 7. Association between TANF policy changes and breastfeeding, alternate identification strategy, restricting sample to income level of $75,000 or less and comparing by education level

eFigure 8. Association between TANF policy changes and breastfeeding, alternate identification strategy, restricting sample to income level of $75,000 or less and comparing by income level

eFigure 9. Association between TANF policy changes and breastfeeding with survey weights

eTable. Details on policy changes for the Temporary Assistance for Needy Families program during the COVID-19 pandemic

eReferences

This supplemental material has been provided by the authors to give readers additional information about their work.

© 2025 Dore EC et al. *JAMA Health Forum*.

eMethods

Data collection and sample

Pregnancy Risk Assessment Monitoring System (PRAMS) surveys are self-administered through a mailed paper questionnaire or interview-administered by telephone. Samples are drawn randomly from a birth certificate file each month, and potential participants are contacted by mail with an invitation to participate within 2 to 6 months after delivery. Mailings are followed up by phone calls, and up to 15 phone calls are made to each participant if nonresponsive.[1] Informed consent was obtained during survey administration.[2]

All states administer questions about breastfeeding, but PRAMS only makes data publicly available for a given state when the response rate meets a given threshold. Seven states did not provide data for the study period and were thus not included: California, Idaho, Nevada, Ohio, South Carolina, Texas, and Vermont. There is no documentation of disruption to data collection during the COVID-19 pandemic. In addition, average participation rates across all sites were similar in 2020 compared to previous years: 56.9% in 2020, 57.0% in 2019, 56.8% in 2018, and 58.0% in 2017.[3]

Variables

Breastfeeding duration was captured as weeks of breastfeeding, coded as zero for those who never breastfed. Of note, the PRAMS survey is administered between two to six months after birth. For those who were still breastfeeding at the time of the survey, we calculated length of breastfeeding as the number of weeks since birth using birth date and survey date. This places a ceiling on breastfeeding duration in the data (i.e., the variable is censored due to the timing of the PRAMS survey), such that actual duration and estimated effects may be larger than what we observed. This is a limitation of PRAMS data, but this technique has been used in prior studies.[4–6]

The composite variable that represents the number of TANF policy changes (results presented in Figure 2) assumes that the marginal TANF policy change is similar for each policy, since we did not weight the policy changes differently when creating the index. Although this is a limitation of the index, there is no a priori reason to weight the policies differently. In addition, the confidence intervals overlap in the analyses examining each individual policy, suggesting equal weighting is appropriate.

Missingness

Missingness for all covariates was less than 1% except for family income, which was 9.2%. Missingness for the two outcomes was 2.8% for breastfeeding initiation and 4.6% for breastfeeding duration. We conducted a complete case analysis since risk of bias is low when missingness is less than 10%, and imputation may add unnecessary noise to the estimates.[7,8]

Main analysis

The equation for the triple-difference analysis we implemented is:

$$\Upsilon_{ist} = \alpha + \beta_1 Policy_{ist}\, x\, During_{ist}\, x\, Lowedu_{ist} + \beta_2 Policy_{ist}\, x\, During_{ist} + \beta_3 Policy_{ist}\, x\, Lowedu_{ist} + \beta_4 During_{ist}\, x\, Lowedu_{ist} + \beta_5 Policy_{ist} + \beta_6 During_{ist} + \beta_7 Lowedu_{ist} + \beta_8 Covars_{ist} + \beta_9 Month_{ist} + \beta_9 State_{ist} + \mathcal{E}_{ist}$$

Υ_{ist} represents the breastfeeding outcome of interest for individual i in state s and month t. $Policy_{ist}$ indicates whether the child was born in a state that implemented the TANF policy change. $During_{ist}$ indicates whether the child was born after the policy was implemented and before it was reversed (when applicable). $Lowedu_{ist}$ indicates whether the mother had a high school education or less, and was therefore a likely TANF participant. We included all two-way and three-way interactions between these three variables. $Covars_{ist}$ represents the maternal individual and state characteristics described in the text. $Month_{ist}$ and $State_{ist}$ represents birth month/year and state fixed effects respectively. β_1 is the coefficient of interest, representing the effect of the TANF policy changes on likely TANF participants.

We estimated linear models for all outcomes, as is standard for difference-in-differences study designs, because of 1) differences in interpretation of interaction terms in non-linear models, and 2) the tendency of logistic models to fail to converge in the setting of many covariates (i.e., state fixed effects).[9,10]

© 2025 Dore EC et al. *JAMA Health Forum*.

eFigure 1. Conceptual diagram linking TANF policy changes to breastfeeding outcomes

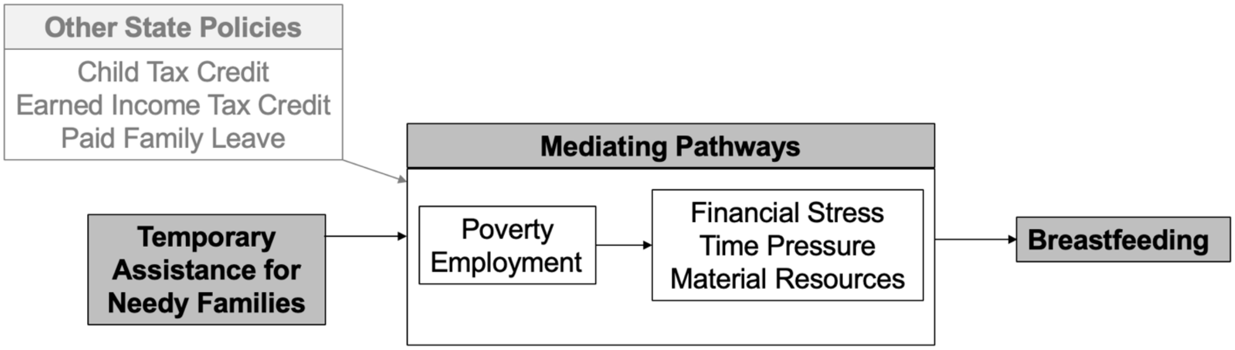

© 2025 Dore EC et al. *JAMA Health Forum*.

eFigure 2. Sample flowchart, Pregnancy Risk Assessment Monitoring System

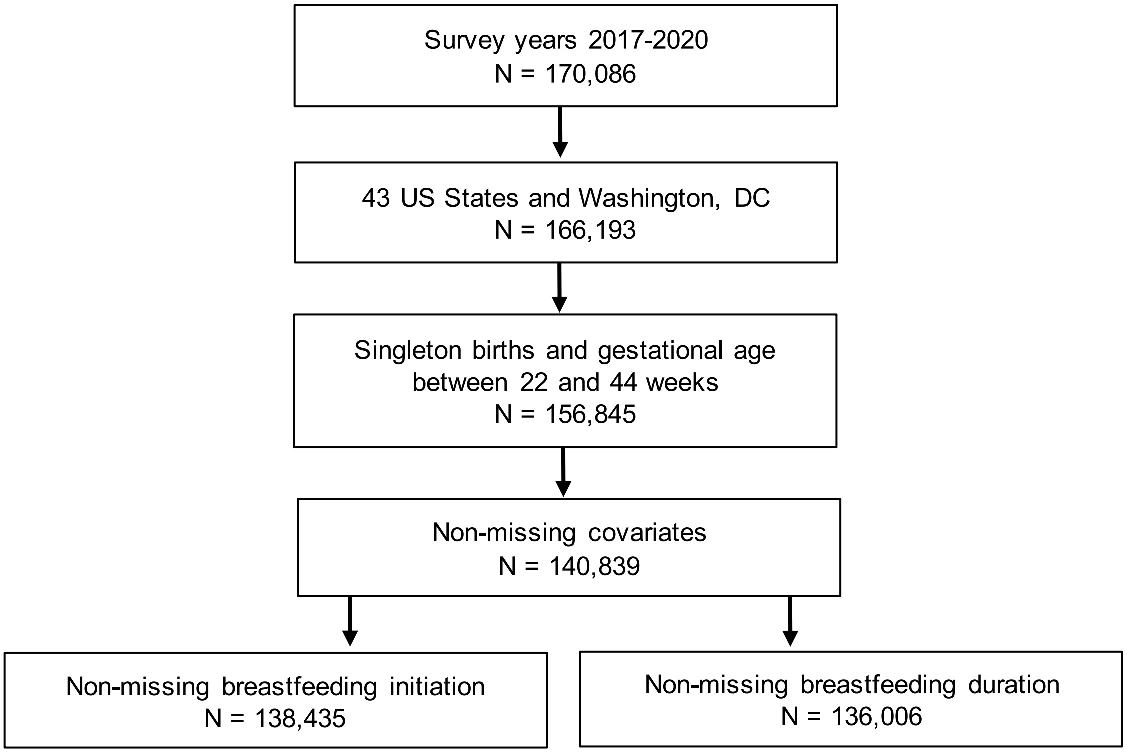

© 2025 Dore EC et al. *JAMA Health Forum*.

eFigure 3. Event study plots for each TANF policy change and breastfeeding initiation

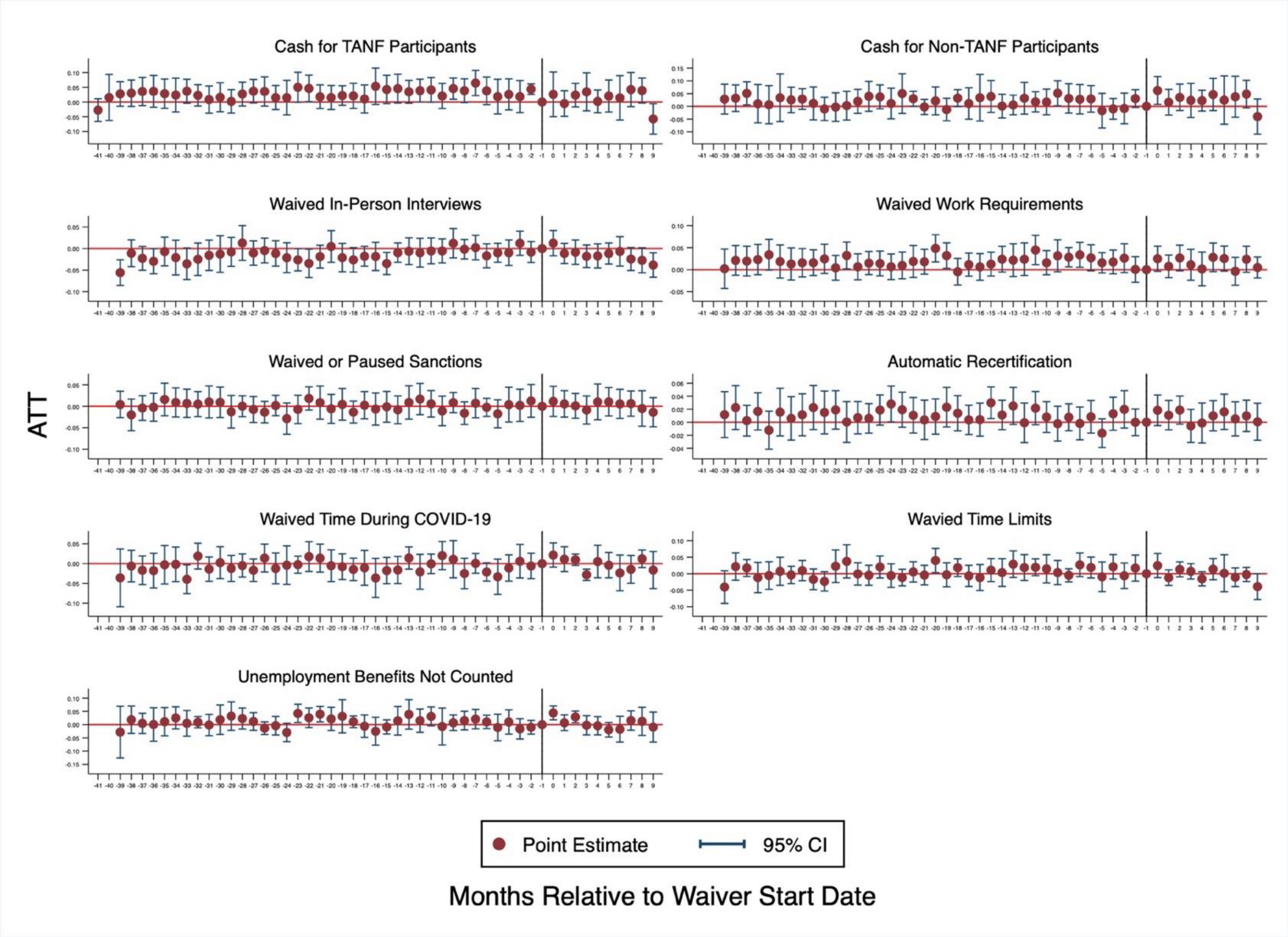

Note: TANF=Temporary Assistance for Needy Families. Each event study plot represents the difference in treatment versus control households (based on whether the individual lived in a state with a policy change or not), before versus after the policy change was implemented. The study period was January 2017-December 2020 and most policy changes were implemented in March or April 2020. Thus, most pre-policy change periods were January 2017-March/April 2020, and during-policy change periods were March/April 2020-December 2020. All event studies adjusted for the other eight policy changes, state-level covariates, maternal characteristics, fixed effects for state and month/year, and clustered standard errors at the state level. N=138,435.

© 2025 Dore EC et al. *JAMA Health Forum*.

eFigure 4. Event study plots for each TANF policy change and breastfeeding duration

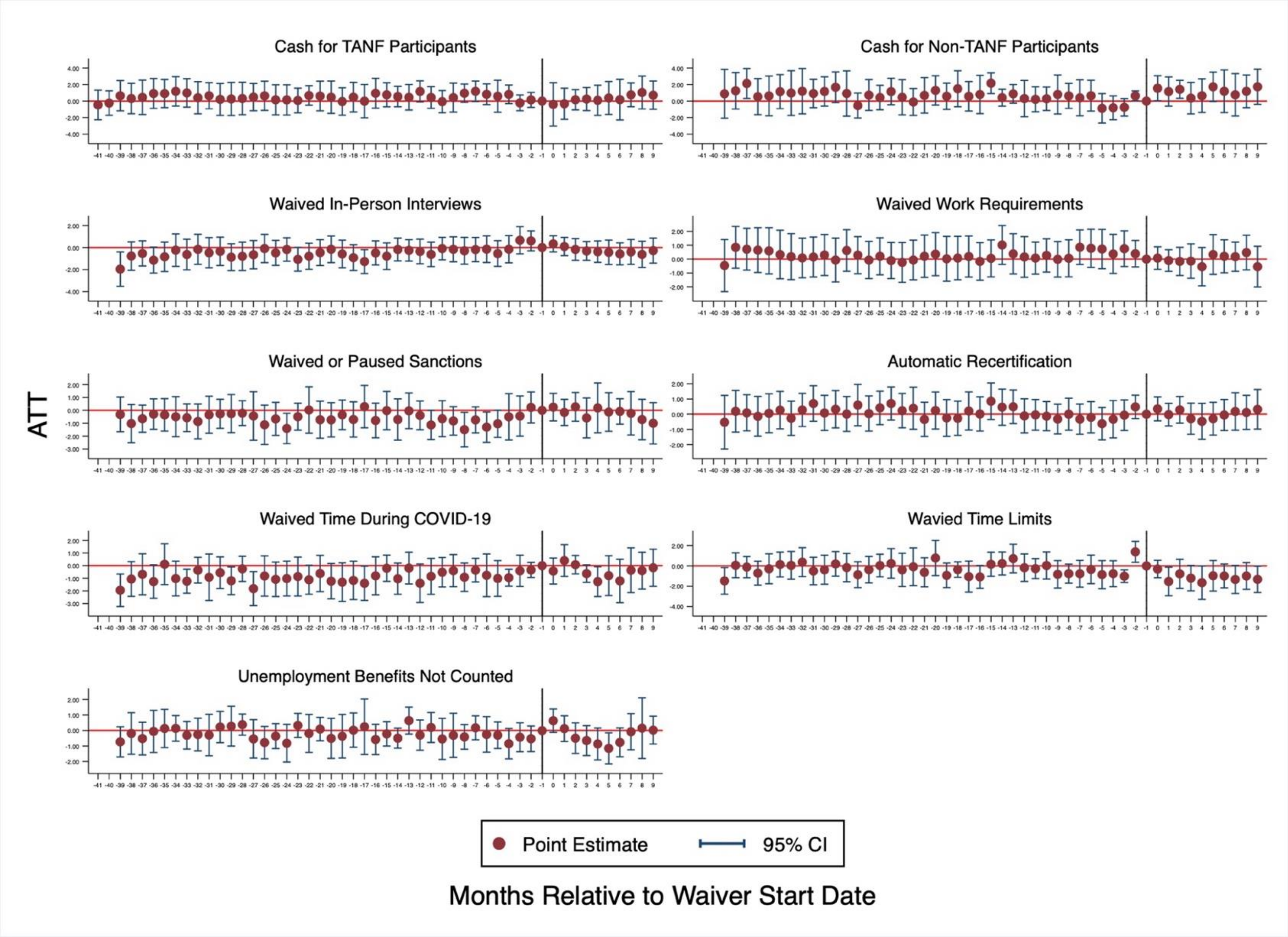

Note: TANF=Temporary Assistance for Needy Families. Each event study plot represents the difference in treatment versus control households (based on whether the individual lived in a state with a policy change or not), before versus after the policy change was implemented. The study period was January 2017-December 2020 and most policy changes were implemented in March or April 2020. Thus, most pre-policy change periods were January 2017-March/April 2020, and during-policy change periods were March/April 2020-December 2020. All event studies adjusted for the other eight policy changes, state-level covariates, maternal characteristics, fixed effects for state and month/year, and clustered standard errors at the state level. N=136,006.

© 2025 Dore EC et al. *JAMA Health Forum*.

eFigure 5. Association between TANF policy changes and breastfeeding initiation by race and ethnicity

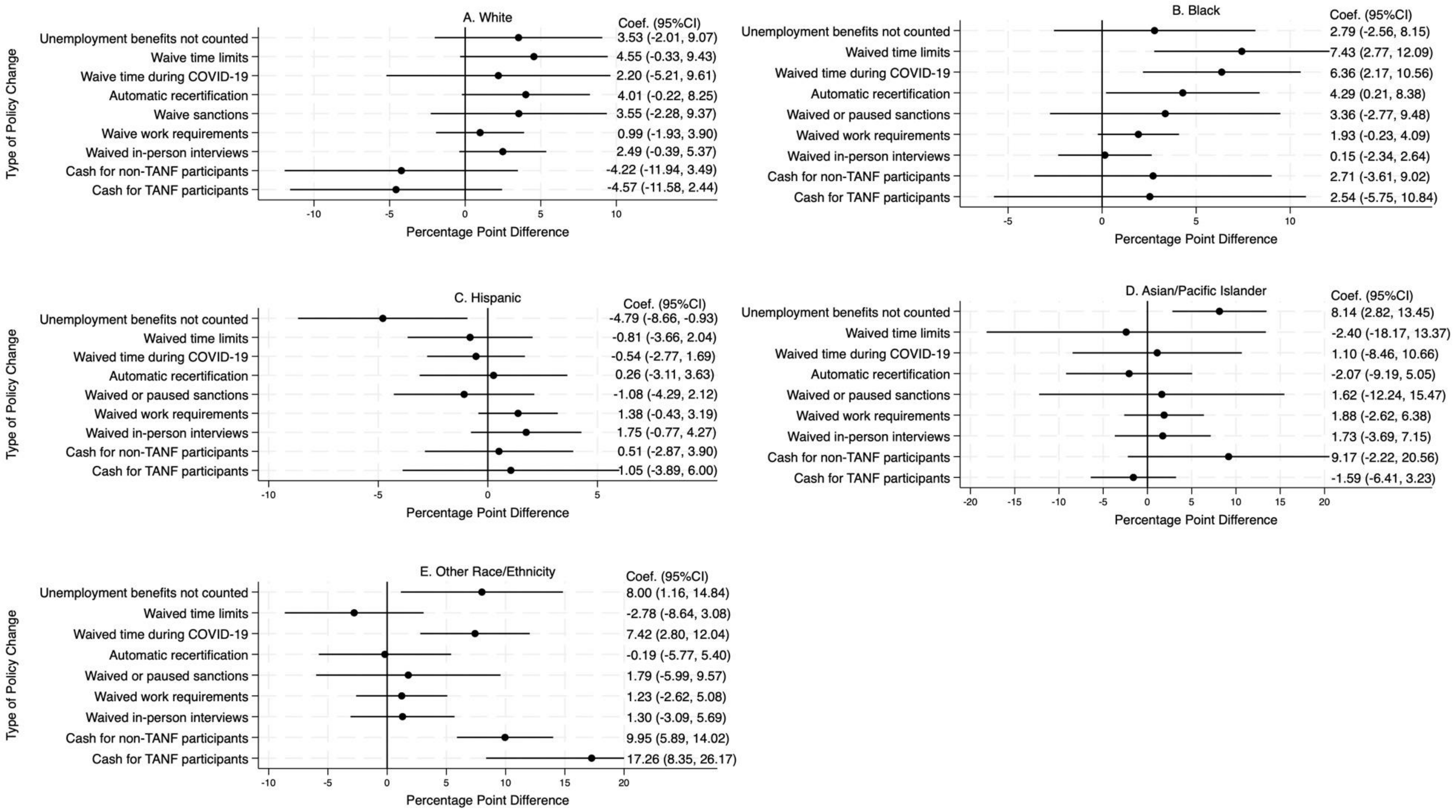

Note: TANF=Temporary Assistance for Needy Families. Results are derived from triple-difference models stratified by race/ethnicity that measured the difference in breastfeeding initiation between likely TANF participants (individuals with high school education or less) and likely nonparticipants (individuals with more than a high school education), in states that had the policy change compared to states that did not have the policy change. All models adjusted for the other eight policy changes, state-level covariates, maternal characteristics, fixed effects for state and month/year, and clustered the standard errors at the state level. Panel A-E shows results for the White sample (N=67,273), Black sample (N=24,955), Hispanic sample (N=22,600), Asian/Pacific Islander sample (N=9,830), and Other race sample (N=13,777) respectively. The Other race group included individuals who identified as multiracial, American Indian or Alaskan Native, or other. The upper confidence interval in Panel E for Cash for TANF participants was truncated to fit, but is 26.17.

© 2025 Dore EC et al. *JAMA Health Forum*.

eFigure 6. Association between TANF policy changes and breastfeeding duration by race and ethnicity

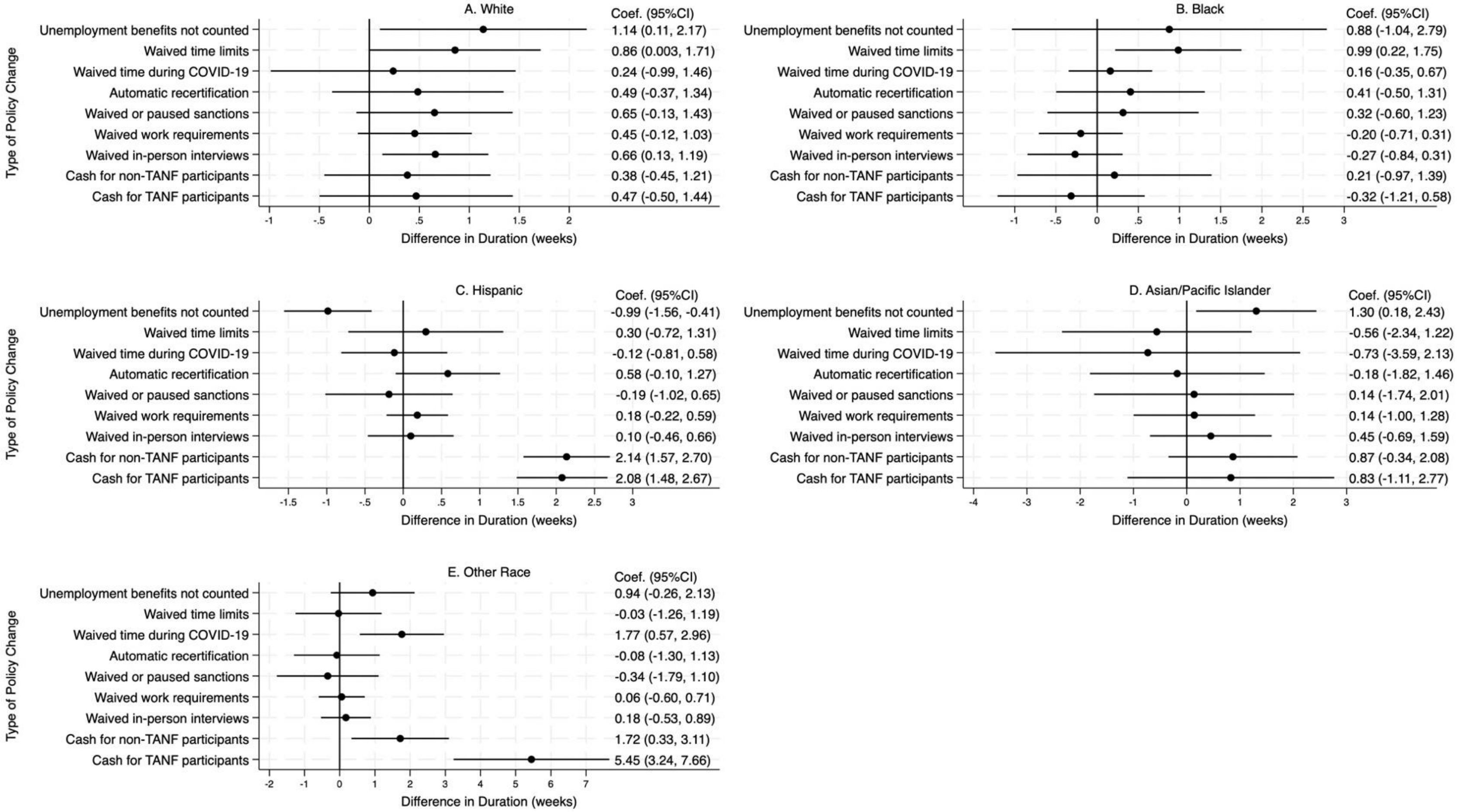

Note: TANF=Temporary Assistance for Needy Families. Results are derived from triple-difference models stratified by race/ethnicity that measured the difference in breastfeeding length between likely TANF participants (individuals with high school education or less) and likely nonparticipants (individuals with more than a high school education), in states that had the policy change compared to states that did not have the policy change. All models adjusted for the other eight policy changes, state-level covariates, maternal characteristics, fixed effects for state and month/year, and clustered the standard errors at the state level. Panel A-E shows results for the White sample (N=66,086), Black sample (N=24,461), Hispanic sample (N=22,276), Asian/Pacific Islander sample (N=9,673), and Other race sample (N=13,510) respectively. The Other race group included individuals who identified as multiracial, American Indian or Alaskan Native, or other.

© 2025 Dore EC et al. *JAMA Health Forum*.

eFigure 7. Association between TANF policy changes and breastfeeding, alternate identification strategy, restricting sample to income level of $75,000 or less and comparing by education level

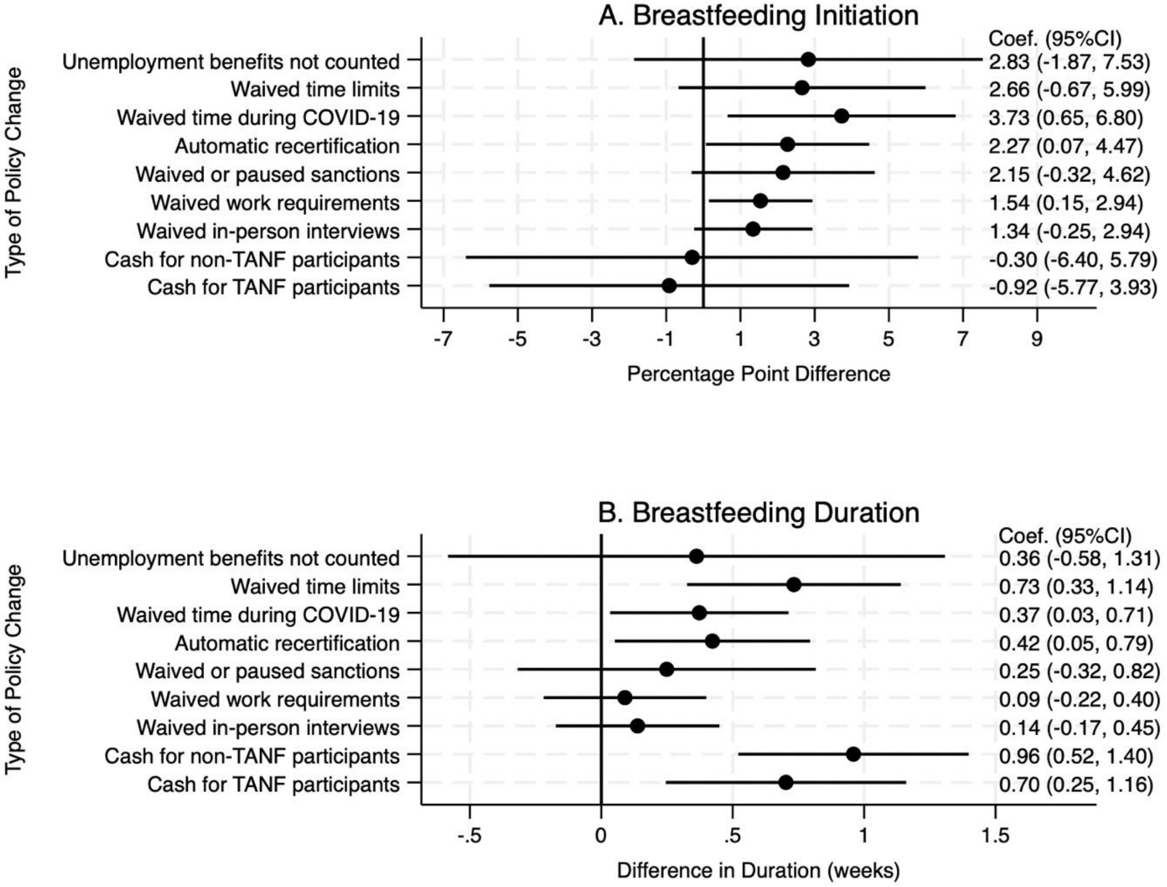

Note: TANF=Temporary Assistance for Needy Families. Panel A N=94,545, Panel B N=92,878. Results are derived from triple-difference models that measured the difference in outcomes in a sample restricted to individuals with yearly incomes of $75,000 or less, which compared likely TANF participants (individuals with high school education or less) and likely non-participants (individuals with more than a high school education), in states that had the policy change compared to states that did not have the policy change. All models adjusted for the other eight policy changes, state-level covariates, maternal characteristics, fixed effects for state and month/year, and clustered the standard errors at the state level.

© 2025 Dore EC et al. *JAMA Health Forum*.

eFigure 8. Association between TANF policy changes and breastfeeding, alternate identification strategy, restricting sample to income level of $75,000 or less and comparing by income level

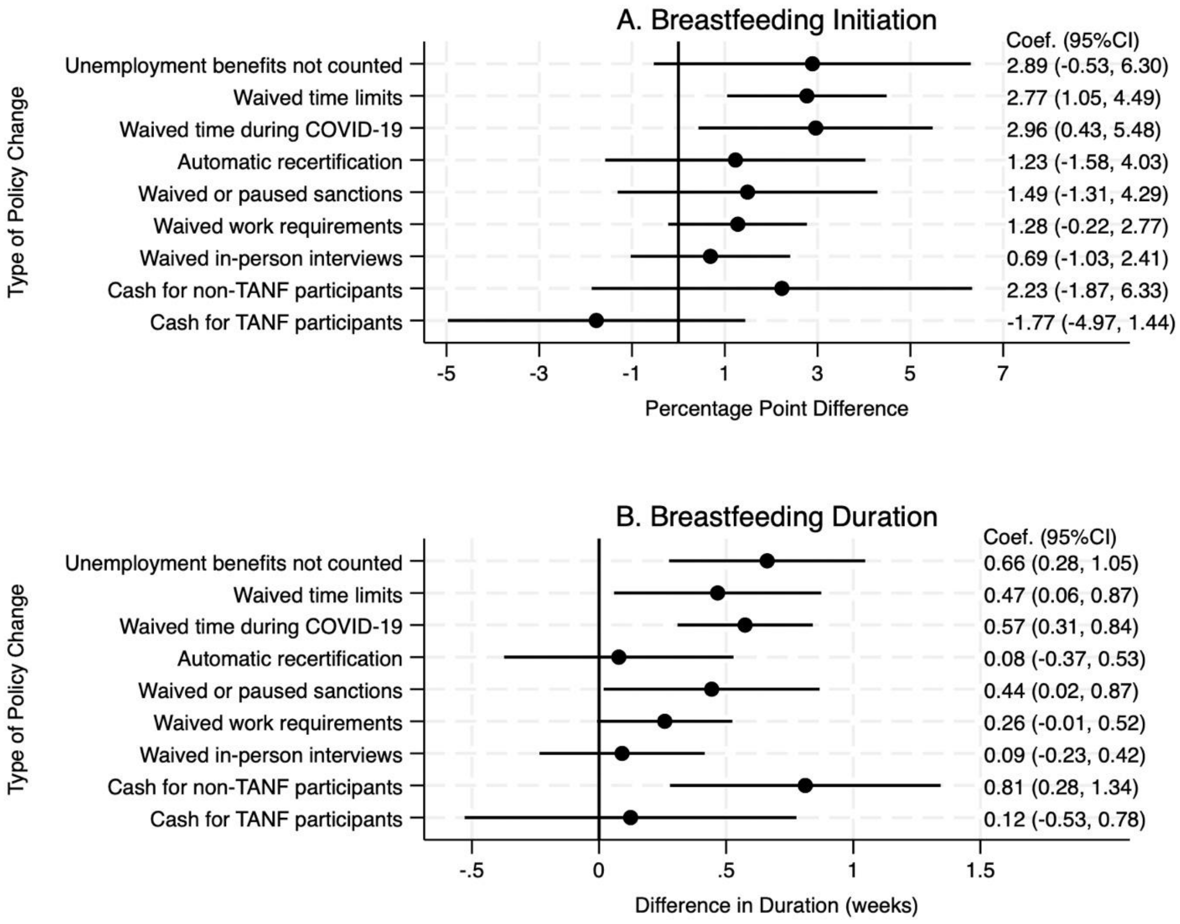

Note: TANF=Temporary Assistance for Needy Families. Panel A N=94,545, Panel B N=92,878. Results are derived from triple-difference models that measured the difference in outcomes in a sample restricted to individuals with yearly incomes of $75,000 or less, which compared likely TANF participants (individuals with yearly income of $25,000 or less) and likely nonparticipants (individuals with yearly income more than $25,000), in states that had the policy change compared to states that did not have the policy change. All models adjusted for the other eight policy changes, state-level covariates, maternal characteristics, fixed effects for state and month/year, and clustered the standard errors at the state level.

© 2025 Dore EC et al. *JAMA Health Forum*.

eFigure 9. Association between TANF policy changes and breastfeeding with survey weights

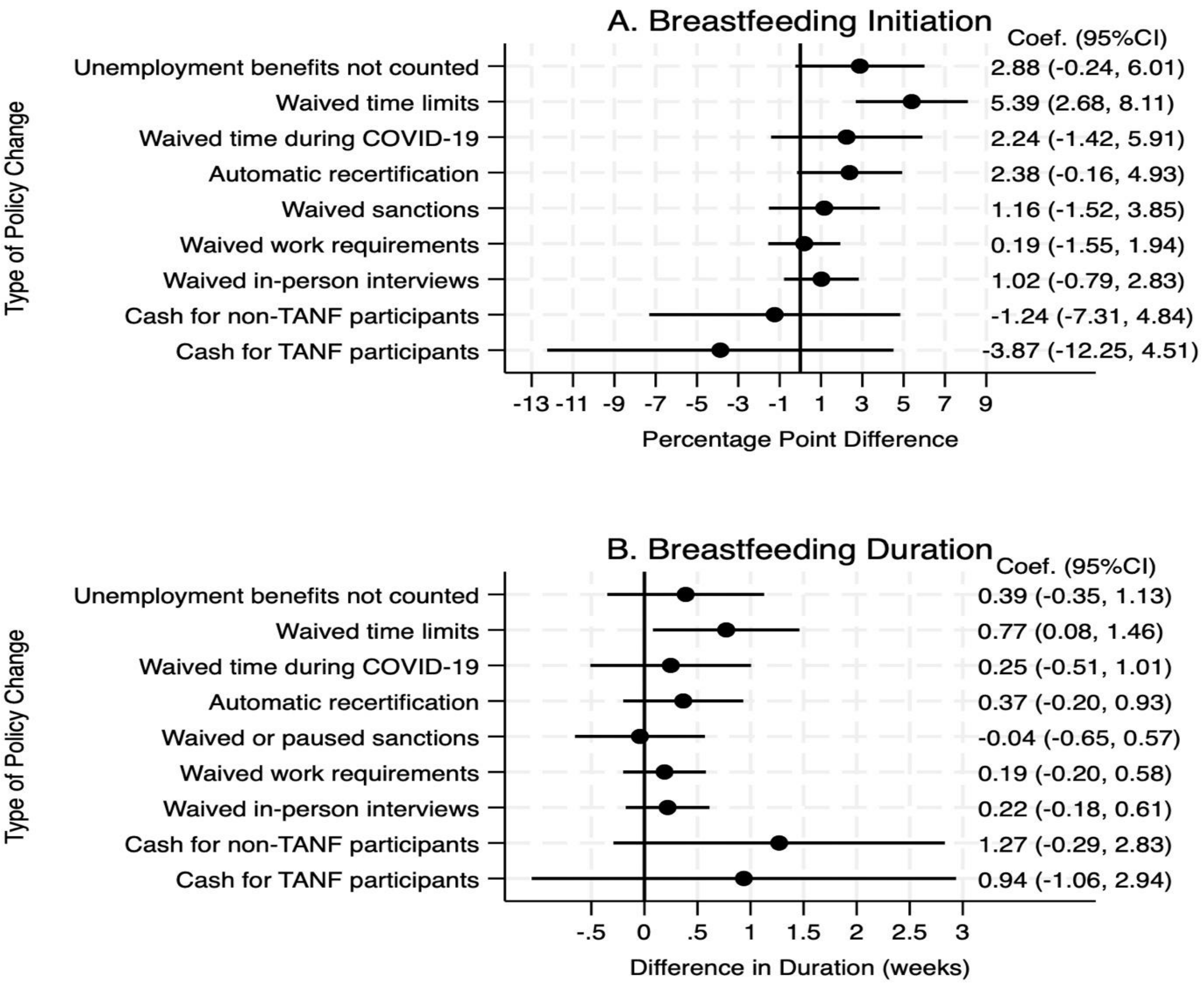

Notes: TANF=Temporary Assistance for Needy Families. Panel A N=138,435, Panel B N=136,006. Results are derived from triple-difference models that measured the difference in outcomes between likely TANF participants (individuals with high school education or less) compared to likely non-participants (individuals with more than a high school education), in states that had the policy change compared to states that did not have the policy change. Each model adjusted for the other eight policy changes, maternal and state-level covariates, and fixed effects for state and month/year. This model also used survey strata and cluster weights to attain estimates.

© 2025 Dore EC et al. *JAMA Health Forum*.

eTable. Details on policy changes for the Temporary Assistance for Needy Families program during the COVID-19 pandemic

Policy Change	Description	States with Policy Change
Unemployment benefits not counted	Did not consider federal pandemic unemployment benefits as income when determining TANF eligibility	California, Delaware, Hawaii, Indiana, New Hampshire, New Mexico, North Carolina
Waived time limits	Allowed participants to continue to participate in TANF when they reached their lifetime limit, and/or allowed participants who had reached the time limit to reapply to participate again	Arizona, Delaware, Maine, Minnesota, New Hampshire, North Carolina, Rhode Island, Washington, Wisconsin
Waived time limits during COVID-19	Did not count time participating in TANF during the pandemic toward the TANF lifetime limit	Alaska, Arizona, California, Connecticut, Delaware, Massachusetts, North Carolina
Automatic recertification	Did not require participants to recertify their eligibility to continue participation	Alaska, Arizona, California, Colorado, Connecticut, Delaware, Florida, Georgia, Hawaii, Indiana, Iowa, Kentucky, Maryland, Massachusetts, Minnesota, New Hampshire, New Jersey, New Mexico, New York, Ohio, Rhode Island, Tennessee, Texas, Virginia, Washington, Wisconsin
Waived sanctions	Allowed participants to continue to receive benefits if they had previously been sanctioned	Arizona, California, Delaware, Georgia, Illinois, Indiana, Maine, New Mexico, New York, North Carolina, Pennsylvania, Washington
Waived work requirements	Did not require participants to work or engage in work-related activities to receive benefits	Alaska, Arizona, Arkansas, California, Colorado, Connecticut, Delaware, Florida, Georgia, Hawaii, Illinois, Indiana, Kentucky, Louisiana, Maine, Maryland, Massachusetts, Michigan, Minnesota, Missouri, Nebraska, New Hampshire, New Jersey, New Mexico, New York, North Carolina, North Dakota, Ohio, Oregon, Pennsylvania, Rhode Island, South Carolina, Texas, Vermont, Virginia, Washington, Wisconsin
Waived in-person interviews	Interviews to determine TANF eligibility were either suspended or held over the phone instead of in-person	Alaska, Arkansas, California, Colorado, Connecticut, Delaware, Florida, Hawaii, Illinois, Kansas, Louisiana, Maine, Maryland, Massachusetts, Michigan, Minnesota, Mississippi, Montana, Nevada, New Hampshire, New Jersey, New York, North Carolina, North Dakota, Ohio, Oklahoma, Oregon, Pennsylvania, Rhode Island, South Carolina, Tennessee, Wisconsin, Wyoming
Cash for non-TANF participants	One-time cash disbursements were given to low-income families who would qualify for TANF but were not TANF participants	Delaware, Virginia, Washington, West Virginia
Cash for TANF participants	One-time additional cash disbursements were given to TANF participants	Alabama, Delaware, Illinois, North Carolina, Oklahoma, Rhode Island, Virginia, West Virginia

Note: TANF=Temporary Assistance for Needy Families.

© 2025 Dore EC et al. *JAMA Health Forum*.

eReferences

1. CDC. Data Methodology. Pregnancy Risk Assessment Monitoring System (PRAMS). February 26, 2025. Accessed April 28, 2025. https://www.cdc.gov/prams/php/methodology/index.html

2. Shulman HB, D'Angelo DV, Harrison L, Smith RA, Warner L. The Pregnancy Risk Assessment Monitoring System (PRAMS): Overview of Design and Methodology. *Am J Public Health*. 2018;108(10):1305-1313. doi:10.2105/AJPH.2018.304563

3. CDC. 2021 PRAMS Response Rate Table. Pregnancy Risk Assessment Monitoring System (PRAMS). May 15, 2024. Accessed March 20, 2025. https://www.cdc.gov/prams/php/data-research/2021-response-rate-table.html

4. Diaz LE, Yee LM, Feinglass J. Rates of breastfeeding initiation and duration in the United States: data insights from the 2016–2019 Pregnancy Risk Assessment Monitoring System. *Front Public Health*. 2023;11:1256432. doi:10.3389/fpubh.2023.1256432

5. Hamad R, Collin DF, Gemmill A, Jackson K, Karasek D. The Pent-Up Demand for Breastfeeding Among US Women: Trends After COVID-19 Shelter-in-Place. *Am J Public Health*. 2023;113(8):870-873. doi:10.2105/AJPH.2023.307313

6. Wells WM, White JS, Collin DF, Wang G, Modrek S, Hamad R. Effects of US state paid family leave policies on perinatal and postpartum health: A quasi-experimental analysis. *Am J Epidemiol*. Published online February 7, 2025:kwaf010. doi:10.1093/aje/kwaf010

7. Langkamp DL, Lehman A, Lemeshow S. Techniques for Handling Missing Data in Secondary Analyses of Large Surveys. *Acad Pediatr*. 2010;10(3):205-210. doi:10.1016/j.acap.2010.01.005

8. Von Hippel PT. Regression with Missing Ys: An Improved Strategy for Analyzing Multiply Imputed Data. *Sociol Methodol*. 2007;37(1):83-117. doi:10.1111/j.1467-9531.2007.00180.x

9. Karaca-Mandic P, Norton EC, Dowd B. Interaction terms in nonlinear models. *Health Serv Res*. 2012;47(1 Pt 1):255-274. doi:10.1111/j.1475-6773.2011.01314.x

10. Athey S, Imbens GW. Identification and Inference in Nonlinear Difference-in-Differences Models. *Econometrica*. 2006;74(2):431-497. doi:10.1111/j.1468-0262.2006.00668.x

© 2025 Dore EC et al. *JAMA Health Forum*.
