# Supplementary material for: Easing Cash Assistance Rules and Breastfeeding
Source: JAMA Health Forum. 2025 Aug 8;6(8):e252999. doi: 10.1001/jamahealthforum.2025.2999 (PMC12334963; doi:10.1001/jamahealthforum.2025.2999)
Supplement: Supplement 2. — Data sharing statement [file jamahealthforum-e252999-s002.pdf]

Data Sharing Statement

Dore. Easing Cash Assistance Rules and Breastfeeding. *JAMA Health Forum*. Published August 08, 2025. doi:10.1001/jamahealthforum.2025.2999

Data
Data available: No

Additional Information
Explanation for why data not available: We used restricted access individual level PRAMS data. The database of TANF policy changes during the COVID-19 pandemic is freely available through the UNC Dataverse: https://doi.org/10.15139/S3/79WVWC
